# Study of coupled nonlinear partial differential equations for finding exact analytical solutions

**DOI:** 10.1098/rsos.140406

**Published:** 2015-07-15

**Authors:** Kamruzzaman Khan, M. Ali Akbar, H. Koppelaar

**Affiliations:** 1 Department of Mathematics, Pabna University of Science and Technology, Pabna 6600, Bangladesh; 2 Department of Applied Mathematics, University of Rajshahi, Rajshahi 6205, Bangladesh; 3 Faculty of Electrical Engineering, Mathematics and Computer Science, University of Delft, Delft, The Netherlands

**Keywords:** enhanced (*G*′/*G*)-expansion method, Drinfel'd–Sokolov–Wilson equation, Painlevé integrable Burgers equation, solitary wave, nonlinear partial differential equations

## Abstract

Exact solutions of nonlinear partial differential equations (NPDEs) are obtained via the enhanced (*G*′/*G*)-expansion method. The method is subsequently applied to find exact solutions of the Drinfel'd–Sokolov–Wilson (DSW) equation and the (2+1)-dimensional Painlevé integrable Burgers (PIB) equation. The efficiency of this method for finding these exact solutions is demonstrated. The method is effective and applicable for many other NPDEs in mathematical physics.

## 1. Introduction

Nonlinear partial differential equations (NPDEs) frequently arise in formulating fundamental laws of nature and in mathematical analysis of a wide variety of problems naturally arising from meteorology, solid-state physics, fluid dynamics, plasma physics, ocean and atmospheric waves, mathematical biology, chemistry, material science, etc. Exact solutions of NPDEs play an important role in the proper understanding of qualitative features of many phenomena and processes in the mentioned areas of natural science. Because exact solutions of nonlinear equations graphically and symbolically substantiate unscrambling the mechanisms of many complex nonlinear phenomena such as spatial localization of transfer processes, multiplicity or absence of steady states under various conditions, existence of peaking regimes and many others. Most physical systems involve several unknown variables and unknown parameters. For example, a system of partial differential equations to describe the motion of a fluid might require density, pressure, temperature and the particle velocity as independent variables. Derived exact analytical travelling wave solutions exhibit solitary waves when special values are given to its unknown parameters. A solitary wave is a localized travelling wave, travelling with constant speed and shape. A soliton is a particular type of solitary wave, which is not destroyed when it collides with another wave of the same kind. Such behaviour is suggested by numerical simulation, but is it really possible that the soliton completely recovers its original shape after a collision? In detailed analysis of the results of such numerical simulations, some ripples can be observed after a collision, and it therefore seems that the original shape is not completely recovered. In order to clarify whether or not solitons are perturbed by collisions, it is necessary to find exact solutions of soliton equations. Generally, it is a very hard task to find exact solutions of NPDEs, including soliton equations. One of the first methods—before computer algebra was born—for finding exact solutions of non-integrable NPDEs was introduced [1] and applied [2] by Kudryashov. However, even if one manages to find a method for solving one nonlinear equation, in general, such a method will not be applicable to other equations. That is, there exists no unified method to solve many types of nonlinear equations.

To remedy this, direct methods have been investigated: exact travelling wave solutions. As a result, many new techniques have been developed by several groups of mathematicians and physicists, such as the simplest equation method [3,4], the modified simple equation method [5–10] building upon Kudryashov's modified simplest equation method [11,12], the tanh-function method [13], the Exp-function method [14,15], the (*G*′/*G*)-expansion method [16–18], the homotopy perturbation method [19,20], the travelling wave hypothesis [21,22], the Tan-Cot function method [23], the enhanced (*G*′/*G*)-expansion method [24,25], the exp⁡(−Φ(ξ))-expansion method [26], so on.

As said, there is no unified method to handle all types of NPDEs. One of the most effective and direct methods for constructing soliton solutions for nonlinear equations is the enhanced (*G*′/*G*)-expansion method [15]. The main idea of this latter method is a retrograde use of Kudryashov's [3,4] original ‘Method of Simplest Equation’. This is explained and applied by Jawad *et al.* [6]. Ample results of this retrograde idea are elucidated by Kudryashov [11,12] and modified in Vitanov [5] and Kudryashov [12]. Our enhanced method [25] also builds upon another idea [10,17,28], by expanding travelling wave solutions of NPDEs as rational functions of (*G*′/*G*), where *G*=*G*(*ξ*) satisfies Kudryashov's [3,4] ‘simplest’ second-order linear ordinary differential equation *G*′′+*μG*=0.

The objective of this paper is to construct by the enhanced (*G*′/*G*)-expansion method *families* of exact solutions for coupled NPDEs in mathematical physics via the Drinfel'd–Sokolov–Wilson (DSW) equation and Painlevé integrable Burgers (PIB) equation.

The paper is organized as follows. In §2, the enhanced (*G*′/*G*)-expansion method is discussed. In §3, we apply this method to the nonlinear evolution equations pointed out above. Section 4 shows the graphical illustration of obtained solutions, and in §5 conclusions are given.

## 2. The enhanced (*G*′/*G*)-expansion method

In this section, we discuss an analytical method, the so-called enhanced (*G*′/*G*)-expansion method, for deriving travelling wave solutions to NPDEs. First, we discuss the method if applied to a problem defined in terms of a NPDE with two independent variables, i.e. one spatial dimension *x* and another the time dimension *t*. Consider the following evolutionary equation for which we find travelling wave solutions:

2.1 $$f(u,u_{t},u_{x},u_{tt},u_{xx},u_{xt},\ldots )=0,\;x\in R\;\text{and}\;t>0,$$

where *u*(*ξ*)=*u*(*x*,*t*) is an unknown function, *f* is a polynomial of *u*(*x*,*t*) and its partial derivatives including the highest order derivatives and nonlinear terms. In the following, we give the main steps of this method [15,16]:

*Step 1.* Combining the independent variables *x* and *t* into one variable *ξ*=*x*±*ωt*, we suppose that
  2.2 u(ξ)=u(x,t),ξ=x±ωt,
  where *ω*∈*R*−{0} is the velocity of the wave.
  The travelling wave transformation equation (2.2) permits us to reduce equation (2.1) to the following ordinary differential equation (ODE):
  2.3 $$g(u,u^{\prime },u^{\prime \prime },\ldots .)=0$$
  where *g* is a polynomial in *u*(*ξ*) and its derivatives, while *u*′(*ξ*)=d*u*/d*ξ*, *u*′′(*ξ*)=d^2^*u*/d*ξ*^2^, so on.
*Step 2.* We suppose that equation (2.3) has the formal solution
  2.4 $$u(\xi )=\sum_{i=-n}^{n}\left(\frac{a_{i}{\left(G^{\prime }/G\right)}^{i}}{{\left(1+\lambda (G^{\prime }/G)\right)}^{i}}+b_{i}{\left(\frac{G^{\prime }}{G}\right)}^{i-1}\sqrt{\sigma \left(1+\frac{{\left(G^{\prime }/G\right)}^{2}}{\mu }\right)}\right),$$
  where *G*=*G*(*ξ*) satisfies the equation
  2.5 $$G^{\prime \prime }+\mu G=0,$$
  with *a*_*i*_,*b*_*i*_(−*n*≤*i*≤*n*;*n*∈*N*), λ is the constant to be determined later and *σ*=±1, *μ*≠0.
*Step 3.* The positive integer *n* can be determined by considering the homogeneous balance between the highest order derivatives and the nonlinear terms appearing in equations (2.1) or (2.3). We denote the degree of *u*(*ξ*) by *D*(*u*(*ξ*))=*n* from which the degrees of other expressions follow:
  2.6 $$D\left(\frac{d^{q}u}{d\xi^{q}}\right)=n+q,\;D\left(u^{p}{\left(\frac{d^{q}u}{d\xi^{q}}\right)}^{s}\right)=np+s(n+q).$$
  Therefore, we can find the value of *n* in equation (2.4), using equation (2.6).
*Step 4.* We substitute equation (2.4) into equation (2.3) using equation (2.5) and then collect all terms of same powers of (*G*′/*G*)^*j*^ and ${\left(G^{\prime }/G\right)}^{j}\sqrt{\sigma (1+(1/\mu ){\left(G^{\prime }/G\right)}^{2})}$ together, then set each coefficient of them to zero to yield an over-determined system of algebraic equations, solving this system for *a*_*i*_,*b*_*i*_(−*n*≤*i*≤*n*;*n*∈*N*), λ and *ω*.
*Step 5.* From the general solution of equation (2.5), we get the following.
  When *μ*<0,
  2.7 $$\frac{G^{\prime }}{G}=\sqrt{-\mu }\tanh\left(\xi_{0}+\sqrt{-\mu \xi }\right)$$
  and
  2.8 $$\frac{G^{\prime }}{G}=\sqrt{-\mu }\coth\left(\xi_{0}+\sqrt{-\mu \xi }\right).$$
  Again, when *μ*>0,
  2.9 $$\frac{G^{\prime }}{G}=\sqrt{\mu }\tan\left(\xi_{0}-\sqrt{\mu \xi }\right)$$
  and
  2.10 $$\frac{G^{\prime }}{G}=\sqrt{\mu }\cot\left(\xi_{0}+\sqrt{\mu \xi }\right),$$
  where *ξ*_0_ is an arbitrary constant. Finally, substituting *a*_*i*_,*b*_*i*_(−*n*≤*i*≤*n*;*n*∈*N*), λ, *ω* and equations (2.7)–(2.10) into equation (2.4), we obtain travelling wave solutions of equation (2.1).

## 3. Application

### (a) The Drinfel'd–Sokolov–Wilson equation

We exemplify the enhanced (*G*′/*G*)-expansion method to find exact solutions and then the solitary wave solutions to the DSW equation in the form

3.1 $$\begin{array}{rl} u_{t}+pvv_{x} & =0\\ \;\mathrm{and}\;\;v_{t}+qv_{xxx}+ruv_{x}+su_{x}v & =0, \end{array}\}$$

where *p*, *q*, *r* and *s* are non-zero parameters.

Suppose a travelling wave transformation equation is

3.2 u(ξ)=u(x,t)andv(ξ)=v(x,t),ξ=x+ωt.

The equation (3.2) transforms equation (3.1) to the following ODEs:

3.3 $$\omega u^{\prime }+pvv^{\prime }=0$$

and

3.4 $$\omega v^{\prime }+qv^{\prime \prime \prime }+ruv^{\prime }+su^{\prime }v=0.$$

By integrating equation (3.3) with respect to *ξ*, and neglecting the constant of integration, we obtain

3.5 $$u=-\frac{pv^{2}}{2\omega }.$$

Substituting equation (3.5) into equation (3.4), we get

3.6 $$2q\omega v^{\prime \prime \prime }+2\omega^{2}v^{\prime }-p(r+2s)v^{2}v^{\prime }=0.$$

Integrating equation (3.6) with respect to **ξ**, we get

3.7 $$2q\omega v^{\prime \prime }+2\omega^{2}v-\frac{p\left(r+2s\right)v^{3}}{3}+C=0,$$

where *C* is a constant of integration.

Now balancing the highest order derivative *v*′′ and nonlinear term *v*^3^ from equation (3.7), we obtain 3*n*=*n*+2, which gives *n*=1.

Hence for *n*=1, equation (2.4) reduces to

3.8 $$\begin{array}{rl} v(\xi ) & =a_{0}+\frac{a_{1}(G^{\prime }/G)}{1+\lambda (G^{\prime }/G)}+\frac{a_{-1}(1+\lambda (G^{\prime }/G))}{\left(G^{\prime }/G\right)}+b_{0}{\left(G^{\prime }/G\right)}^{-1}\sqrt{\sigma \left(1+\frac{{\left(G^{\prime }/G\right)}^{2}}{\mu }\right)}\\ & \;+b_{1}\sqrt{\sigma \left(1+\frac{{\left(G^{\prime }/G\right)}^{2}}{\mu }\right)}+b_{-1}{\left(G^{\prime }/G\right)}^{-2}\sqrt{\sigma \left(1+\frac{{\left(G^{\prime }/G\right)}^{2}}{\mu }\right)}, \end{array}$$

where *G*=*G*(*ξ*) satisfies equation (2.5). By substituting equation (3.8) along with equation (2.5) into equation (3.7), we get a polynomial of (*G*′/*G*)^*j*^ and ${\left(G^{\prime }/G\right)}^{j}\sqrt{\sigma (1+{\left(G^{\prime }/G\right)}^{2}/\mu )}$. From this polynomial, we equate the coefficients of these two terms and set them to zero. We obtain an over-determined system consisting of 25 algebraic equations. Solving this system for *a*_*i*_, *b*_*i*_, λ and *ω* yields the following values with the aid of symbolic computer software Maple 13.

$$\begin{array}{rl} \mathrm{Set}\;1:\; & C=0,\;\omega =-8q\mu ,\;\lambda =0,\;a_{-1}=\pm \frac{\mu q}{4}\sqrt{\left(\frac{-6\mu }{p(2s+r)}\right)},\;a_{0}=0,\;a_{1}=\mp 4q\sqrt{\left(\frac{-6\mu }{p(2s+r)}\right)},\\ & b_{-1}=b_{0}=b_{1}=0.\\ \mathrm{Set}\;2:\; & C=0,\;\omega =4q\mu ,\;\lambda =0,\;a_{-1}=\pm \frac{\mu q}{4}\sqrt{\left(\frac{3\mu }{p(2s+r)}\right)},\;a_{0}=0,\;a_{1}=\mp 4q\sqrt{\left(\frac{3\mu }{p(2s+r)}\right)},\\ & b_{-1}=b_{0}=b_{1}=0.\\ \mathrm{Set}\;3:\; & C=0,\;\omega =-2q\mu ,\;\lambda =0,\;a_{-1}=0,\;a_{0}=0,\;a_{1}=\mp 2q\sqrt{\left(\frac{-6\mu }{p(2s+r)}\right)},\;b_{-1}=b_{0}=b_{1}=0.\\ \mathrm{Set}\;4:\; & C=0,\;\omega =-\frac{1}{2}q\mu ,\;\lambda =0,\;a_{-1}=0,\;a_{0}=0,\;a_{1}=\pm \frac{1}{2}q\sqrt{\left(\frac{-6\mu }{p(2s+r)}\right)},\;b_{-1}=b_{0}=0,\\ & b_{1}=\mp \frac{3\mu q}{\sqrt{-6\sigma p(2s+r)}}.\\ \mathrm{Set}\;5:\; & C=0,\;\omega =-2q\mu ,\;\lambda =\lambda ,\;a_{-1}=\pm 2\mu q\sqrt{\left(\frac{-6\mu }{p(2s+r)}\right)},\;a_{0}=\mp 2\mu \lambda q\sqrt{\left(\frac{-6\mu }{p(2s+r)}\right)},\\ & a_{1}=b_{-1}=b_{0}=b_{1}=0.\\ \mathrm{Set}\;6:\; & C=0,\;\omega =q\mu ,\;\lambda =\lambda ,\;a_{-1}=a_{0}=a_{1}=b_{-1}=b_{0}=0,\;b_{1}=\pm \frac{6\mu q}{\sqrt{3\sigma p(2s+r)}}.\\ \mathrm{Set}\;7:\; & C=0,\;\omega =-2q\mu ,\;\lambda =\pm \frac{a_{0}}{12\mu^{2}q}\sqrt{-6\mu p(2s+r)},\;a_{-1}=0,\;a_{0}=a_{0},\\ & a_{1}=\pm \frac{24q^{2}\mu^{2}-pra_{0}^{2}-2psa_{0}^{2}}{\mu^{2}q}\sqrt{\left(\frac{-6\mu }{p(2s+r)}\right)}b_{-1}=b_{0}=b_{1}=0.\\ \mathrm{Set}\;8:\; & C=0,\;\omega =q\mu ,\;\lambda =\lambda ,\;a_{-1}=a_{0}=a_{1}=b_{-1}=0,\;b_{0}=\mp 2\mu q\sqrt{\left(\frac{\mu }{p\sigma (2s+r)}\right)},\;b_{1}=0.\\ \mathrm{Set}\;9:\; & C=0,\;\omega =-\frac{1}{2}q\mu ,\;\lambda =0,\;a_{-1}=\pm \frac{1}{2}\mu q\sqrt{\left(\frac{-6\mu }{p(2s+r)}\right)},\;a_{0}=a_{1}=b_{-1}=0,\\ & b_{0}=\pm \frac{1}{2}\mu q\sqrt{\left(\frac{-6\mu }{p\sigma (2s+r)}\right)},\;b_{1}=0. \end{array}$$

By substituting Set 1–Set 9 along with equations (2.7)–(2.10) into equation (3.8), we deduce abundant travelling wave solutions of equation (3.1) as follows.

If *μ*<0, we get the following hyperbolic solutions:

$$\begin{array}{rl} \mathrm{Family}\;1:\; & v_{1}(\xi )=\mp 8\mu q\sqrt{\left(\frac{6}{p(2s+r)}\right)}\coth\left(2\left(\xi_{0}+\sqrt{-\mu }\xi \right)\right),\\ & u_{1}(\xi )=\frac{24\mu q}{2s+r}\coth^{2}\left(2\left(\xi_{0}+\sqrt{-\mu }\xi \right)\right), \end{array}$$

where *ξ*=*x*−8*qμt*.

$$\begin{array}{rl} \mathrm{Family}\;2:\; & v_{2}(\xi )=\mp 8\mu q\sqrt{\frac{-3}{p(2s+r)}}\mathrm{csch}\left(2\left(\xi_{0}+\sqrt{-\mu }\xi \right)\right),\\ & v_{3}(\xi )=\mp I8\mu q\sqrt{\frac{-3}{p(2s+r)}}\mathrm{sech}\left(2\left(\xi_{0}+\sqrt{-\mu }\xi \right)\right),\\ & u_{2}(\xi )=\frac{24q\mu }{2s+r}{\mathrm{csch}}^{2}\left(2\left(\xi_{0}+\sqrt{-\mu }\xi \right)\right),\\ & u_{3}(\xi )=-\frac{24q\mu }{2s+r}{\mathrm{sech}}^{2}\left(2\left(\xi_{0}+\sqrt{-\mu }\xi \right)\right), \end{array}$$

where *ξ*=*x*+4*μqt*.

$$\begin{array}{rl} \mathrm{Family}\;3:\; & v_{4}(\xi )=\pm 2\mu q\sqrt{\frac{6}{p(2s+r)}}\tanh\left(\xi_{0}+\sqrt{-\mu }\xi \right),\\ & v_{5}(\xi )=\pm 2\mu q\sqrt{\frac{6}{p(2s+r)}}\coth\left(\xi_{0}+\sqrt{-\mu }\xi \right),\\ & u_{4}(\xi )=\frac{6\mu q}{2s+r}\tanh^{2}\left(\xi_{0}+\sqrt{-\mu }\xi \right),\\ & u_{5}(\xi )=\frac{6\mu q}{2s+r}\coth^{2}\left(\xi_{0}+\sqrt{-\mu }\xi \right), \end{array}$$

where *ξ*=*x*−2*qμt*.

$$\begin{array}{rl} \mathrm{Family}\;4:\; & v_{6}(\xi )=\pm \frac{\mu q}{2}\sqrt{\frac{6}{p(2s+r)}}\left(\tanh\left(\xi_{0}+\sqrt{-\mu }\xi \right)\pm \mathrm{Isech}\left(\xi_{0}+\sqrt{-\mu }\xi \right)\right),\\ & v_{7}(\xi )==\pm \frac{\mu q}{2}\sqrt{\frac{6}{p(2s+r)}}\left(\coth\left(\xi_{0}+\sqrt{-\mu }\xi \right)\pm \mathrm{csch}\left(\xi_{0}+\sqrt{-\mu }\xi \right)\right),\\ & u_{6}(\xi )=\frac{3\mu q}{2(2s+r)}{\left(\tanh\left(\xi_{0}+\sqrt{-\mu }\xi \right)\pm \mathrm{Isech}\left(\xi_{0}+\sqrt{-\mu }\xi \right)\right)}^{2},\\ & u_{7}(\xi )=\frac{3\mu q}{2(2s+r)}{\left(\coth\left(\xi_{0}+\sqrt{-\mu }\xi \right)\pm \mathrm{csch}\left(\xi_{0}+\sqrt{-\mu }\xi \right)\right)}^{2}, \end{array}$$

where $\xi =x-\frac{1}{2}q\mu t$.

$$\begin{array}{rl} \mathrm{Family}\;5:\; & v_{8}(\xi )=\mp 2\mu q\sqrt{\frac{6}{p(2s+r)}}\coth\left(\xi_{0}+\sqrt{-\mu }\xi \right),\\ & v_{9}(\xi )=\mp 2\mu q\sqrt{\frac{6}{p(2s+r)}}\tanh\left(\xi_{0}+\sqrt{-\mu }\xi \right),\\ & u_{8}(\xi )=\frac{6\mu q}{2s+r}\coth^{2}\left(\xi_{0}+\sqrt{-\mu }\xi \right),\\ & u_{9}(\xi )=\frac{6\mu q}{2s+r}\tanh^{2}\left(\xi_{0}+\sqrt{-\mu }\xi \right), \end{array}$$

where *ξ*=*x*−2*qμt*.

$$\begin{array}{rl} \mathrm{Family}\;6:\; & v_{10}(\xi )=\pm 2\mu q\sqrt{\frac{3}{p(2s+r)}}\mathrm{sech}\left(\xi_{0}+\sqrt{-\mu }\xi \right),\\ & v_{11}(\xi )=\pm I2\mu q\sqrt{\frac{3}{p(2s+r)}}\mathrm{csch}\left(\xi_{0}+\sqrt{-\mu }\xi \right),\\ & u_{10}(\xi )=\frac{6\mu q}{2s+r}{\mathrm{sech}}^{2}\left(\xi_{0}+\sqrt{-\mu }\xi \right),\\ & u_{11}(\xi )=\frac{6\mu q}{2s+r}{\mathrm{csch}}^{2}\left(\xi_{0}+\sqrt{-\mu }\xi \right), \end{array}$$

where *ξ*=*x*+*qμt*.

$$\begin{array}{rl} \mathrm{Family}\;7:\; & v_{12}(\xi )=\pm \frac{12\mu q}{\sqrt{6A}}\left(\frac{a_{0}-2\mu q\sqrt{\frac{6}{A}}\tanh\left(\xi_{0}+\sqrt{-\mu }\xi \right)}{2\mu q\sqrt{\frac{6}{A}}-a_{0}\tanh\left(\xi_{0}+\sqrt{-\mu }\xi \right)}\right),\\ & v_{13}(\xi )=\pm \frac{12\mu q}{\sqrt{6A}}\left(\frac{a_{0}-2\mu q\sqrt{\frac{6}{A}}\coth\left(\xi_{0}+\sqrt{-\mu }\xi \right)}{2\mu q\sqrt{\frac{6}{A}}-a_{0}\coth\left(\xi_{0}+\sqrt{-\mu }\xi \right)}\right),\\ & u_{12}(\xi )=\frac{6pq\mu }{A}{\left(\frac{a_{0}-2\mu q\sqrt{\frac{6}{A}}\tanh\left(\xi_{0}+\sqrt{-\mu }\xi \right)}{2\mu q\sqrt{\frac{6}{A}}-a_{0}\tanh\left(\xi_{0}+\sqrt{-\mu }\xi \right)}\right)}^{2},\\ & u_{13}(\xi )=\frac{6pq\mu }{A}{\left(\frac{a_{0}-2\mu q\sqrt{\frac{6}{A}}\coth\left(\xi_{0}+\sqrt{-\mu }\xi \right)}{2\mu q\sqrt{\frac{6}{A}}-a_{0}\coth\left(\xi_{0}+\sqrt{-\mu }\xi \right)}\right)}^{2}, \end{array}$$

where *A*=*p*(2*s*+*r*), *ξ*=*x*−2*qμt* and $a_{0}\neq 2\mu q\sqrt{6/A}$.

$$\begin{array}{rl} \mathrm{Family}\;8:\; & v_{14}(\xi )=\mp 2q\mu \sqrt{\frac{-3}{p\left(2s+r\right)}}\mathrm{csch}\left(\xi_{0}+\sqrt{-\mu }\xi \right),\\ & v_{15}(\xi )=\mp I2q\mu \sqrt{\frac{-3}{p\left(2s+r\right)}}\mathrm{sech}\left(\xi_{0}+\sqrt{-\mu }\xi \right),\\ & u_{14}(\xi )=\frac{6\mu q}{2s+r}{\mathrm{csch}}^{2}\left(\xi_{0}+\sqrt{-\mu }\xi \right),\\ & u_{15}(\xi )=-\frac{6\mu q}{2s+r}{\mathrm{sech}}^{2}\left(\xi_{0}+\sqrt{-\mu }\xi \right), \end{array}$$

where *ξ*=*x*+*qμt*.

$$\begin{array}{rl} \mathrm{Family}\;9:\; & v_{16}(\xi )=\pm \frac{\mu q}{2}\sqrt{\frac{6}{p(2s+r)}}\left(\coth\left(\xi_{0}+\sqrt{-\mu }\xi \right)\pm \mathrm{csch}\left(\xi_{0}+\sqrt{-\mu }\xi \right)\right),\\ & v_{17}(\xi )=\pm \frac{\mu q}{2}\sqrt{\frac{6}{p(2s+r)}}\left(\tanh\left(\xi_{0}+\sqrt{-\mu }\xi \right)\pm \mathrm{Isech}\left(\xi_{0}+\sqrt{-\mu }\xi \right)\right),\\ & u_{16}(\xi )=\frac{3\mu q}{2(2s+r)}{\left(\coth\left(\xi_{0}+\sqrt{-\mu }\xi \right)\pm \mathrm{csch}\left(\xi_{0}+\sqrt{-\mu }\xi \right)\right)}^{2},\\ & u_{17}(\xi )=\frac{3\mu q}{2(2s+r)}{\left(\tanh\left(\xi_{0}+\sqrt{-\mu }\xi \right)\pm \mathrm{Isech}\left(\xi_{0}+\sqrt{-\mu }\xi \right)\right)}^{2}, \end{array}$$

where *ξ*=*x*−1/2*qμt*.

Consequently, for *μ*>0, we can obtain nine corresponding families of plane periodic solutions (which are omitted for convenience).

**Note**

From the obtained solutions we observe that *r*≠−2*s*.

**Remark 3.1**

All the obtained results have been checked with Maple 13 by putting them back into the original equation. All results are correct.

### (b) The (2+1)-dimensional Painlevé integrable Burgers equation

In this subsection, we will exert the enhanced (*G*′/*G*)-expansion method to find the exact solution and then the solitary wave solutions of the PIB equation in the form,

3.9 $$-u_{t}+uu_{y}+\alpha vu_{x}+\beta u_{yy}+\alpha \beta u_{xx}=0$$

and

3.10 $$u_{x}-v_{y}=0.$$

where *α* and *β* are non-zero constants. This system of equations was derived from the generalized Painlevé integrability classification.

The travelling wave transformation equation

3.11 u(ξ)=u(x,y,t)andv(ξ)=v(x,y,t),ξ=x+y−ωt,

permits us to transform equations (3.9) and (3.10) to the following ODEs:

3.12 $$\omega u^{\prime }+uu^{\prime }+\alpha vu^{\prime }+\beta u^{\prime \prime }+\alpha \beta u^{\prime \prime }=0$$

and

3.13 $$u^{\prime }-v^{\prime }=0.$$

Integrating equation (3.13) with respect to **ξ**, we get

3.14 v=u+R,

where *R* is a constant of integration.

Substituting equation (3.14) into equation (3.12), and then integrating with respect to *ξ*, setting constant of integration to zero, yields

3.15 $$(\omega +\alpha R)u+\frac{1}{2}(\alpha +1)u^{2}+\beta (\alpha +1)u^{\prime }=0.$$

Balancing the highest order derivative *u*′ and nonlinear term *u*^2^ from equation (3.15), we obtain 2*n*=*n*+1, which gives *n*=1.

Hence for *n*=1, equation (2.4) reduces to

3.16 $$\begin{array}{rl} u(\xi ) & =a_{0}+\frac{a_{1}(G^{\prime }/G)}{1+\lambda (G^{\prime }/G)}+\frac{a_{-1}(1+\lambda (G^{\prime }/G))}{\left(G^{\prime }/G\right)}+b_{0}{\left(\frac{G^{\prime }}{G}\right)}^{-1}\sqrt{\sigma \left(1+\frac{{\left(G^{\prime }/G\right)}^{2}}{\mu }\right)}\\ & \;+b_{1}\sqrt{\sigma \left(1+\frac{{\left(G^{\prime }/G\right)}^{2}}{\mu }\right)}+b_{-1}{\left(G^{\prime }/G\right)}^{-2}\sqrt{\sigma \left(1+\frac{{\left(G^{\prime }/G\right)}^{2}}{\mu }\right)}, \end{array}$$

where *G*=*G*(*ξ*) satisfies equation (2.5). Substituting equation (3.6) along with equation (2.5) into equation (3.15), we get a polynomial of (*G*′/*G*)^*j*^ and ${\left(G^{\prime }/G\right)}^{j}\sqrt{\sigma (1+{\left(G^{\prime }/G\right)}^{2}/\mu )}$. From this polynomial, we equate the coefficients of (*G*′/*G*)^*j*^ and ${\left(G^{\prime }/G\right)}^{j}\sqrt{\sigma (1+{\left(G^{\prime }/G\right)}^{2}/\mu )}$ and setting them to zero we get an over-determined system that consists of 25 algebraic equations. Solving this system for *a*_*i*_,*b*_*i*_,λ and *ω*, we obtain the following values with the aid of symbolic computer software Maple 13.

$$\begin{array}{rl} \mathrm{Set}\;1:\; & R=R,\;S=2a_{0}\beta \alpha \mu \lambda +\frac{1}{2}\alpha a_{0}^{2}+2a_{0}\beta \mu \lambda +\frac{1}{2}a_{0}^{2}+2\beta^{2}\alpha \mu^{2}\lambda^{2}+2\alpha \beta^{2}\mu +2\beta^{2}\mu^{2}\lambda^{2}+2\beta^{2}\mu ,\\ & \omega =-2\beta \mu \lambda -2\beta \alpha \mu \lambda -a_{0}-\alpha R-\alpha a_{0},\;\lambda =\lambda ,\;a_{-1}=0,\;a_{0}=a_{0},\;a_{1}=2\beta \mu \lambda^{2}+2\beta ,\\ & b_{-1}=0,\;b_{0}=0,\;b_{1}=0.\\ \mathrm{Set}\;2:\; & R=R,\;S=2\beta^{2}\mu^{2}\lambda^{2}+2\beta^{2}\alpha \mu^{2}\lambda^{2}-2a_{0}\beta \mu \lambda -2a_{0}\beta \alpha \mu \lambda +\frac{1}{2}a_{0}^{2}+\frac{1}{2}\alpha a_{0}^{2}+2\alpha \beta^{2}\mu +2\beta^{2}\mu ,\\ & \omega =2\beta \mu \lambda +2\beta \alpha \mu \lambda -a_{0}-\alpha R-\alpha a_{0},\;\lambda =\lambda ,\;a_{-1}=-2\beta \mu ,\;a_{0}=a_{0},\\ & a_{1}=0,\;b_{-1}=0,\;b_{0}=0,\;b_{1}=0.\\ \mathrm{Set}\;3:\; & R=R,\;S=\frac{1}{2}a_{0}^{2}+\frac{1}{2}\alpha a_{0}^{2}+8\alpha \beta^{2}\mu +8\beta^{2}\mu ,\;\omega =-a_{0}-\alpha R-\alpha a_{0},\;\lambda =0,\;a_{-1}=-2\beta \mu ,\\ & a_{0}=a_{0},\;a_{1}=2\beta ,\;b_{-1}=0,\;b_{0}=0,\;b_{1}=0.\\ \mathrm{Set}\;4:\; & R=R,\;S=\frac{1}{2}(\beta^{2}\mu^{2}\lambda^{2}+a_{0}^{2}-2a_{0}\beta \mu \lambda +\beta^{2}\mu )(1+\alpha ),\;\omega =\beta \mu \lambda +\beta \alpha \mu \lambda -a_{0}-\alpha R-\alpha a_{0},\\ & \lambda =\lambda ,\;a_{-1}=-\beta \mu ,\;a_{0}=a_{0},\;a_{1}=0,\;b_{-1}=0,\;b_{0}=\pm \frac{\beta \mu }{\sqrt{\sigma }},\;b_{1}=0.\\ \mathrm{Set}\;5:\; & R=R,\;S=\frac{1}{2}(a_{0}^{2}+\beta^{2}\mu )\left(1+\alpha \right),\;\omega =-a_{0}-\alpha R-\alpha a_{0},\;\lambda =0,\;a_{-1}=0,\;a_{0}=a_{0},\;a_{1}=\beta ,\\ & b_{-1}=0,\;b_{0}=0,\;b_{1}=\pm \beta \sqrt{\frac{\mu }{\sigma }}. \end{array}$$

For the values of above sets we obtain the following travelling wave solutions for PIB equations.

Hyperbolic function solutions: for *μ*<0

$$\begin{array}{rl} \mathrm{Family}\;1:\; & u_{1}(\xi )=a_{0}+2\beta (1+\mu \lambda^{2})\sqrt{-\mu }{\left(\coth\left(\xi_{0}+\sqrt{-\mu }\xi \right)+\lambda \sqrt{-\mu }\right)}^{-1},\\ & u_{2}(\xi )=a_{0}+2\beta (1+\mu \lambda^{2})\sqrt{-\mu }{\left(\tanh\left(\xi_{0}+\sqrt{-\mu }\xi \right)+\lambda \sqrt{-\mu }\right)}^{-1},\\ & v_{1}(\xi )=a_{0}+2\beta (1+\mu \lambda^{2})\sqrt{-\mu }{\left(\coth\left(\xi_{0}+\sqrt{-\mu }\xi \right)+\lambda \sqrt{-\mu }\right)}^{-1}+R,\\ & v_{2}(\xi )=a_{0}+2\beta (1+\mu \lambda^{2})\sqrt{-\mu }{\left(\tanh\left(\xi_{0}+\sqrt{-\mu }\xi \right)+\lambda \sqrt{-\mu }\right)}^{-1}+R, \end{array}$$

where *ξ*=*x*+*y*+(2*βμ*λ+2*βαμ*λ+*a*_0_+*αR*+*αa*_0_)*t*.

$$\begin{array}{rl} \mathrm{Family}\;2:\; & u_{3}(\xi )=a_{0}+2\beta \sqrt{-\mu }\left(\coth\left(\xi_{0}+\sqrt{-\mu }\xi \right)+\lambda \sqrt{-\mu }\right),\\ & u_{4}(\xi )=a_{0}+2\beta \sqrt{-\mu }\left(\tanh\left(\xi_{0}+\sqrt{-\mu }\xi \right)+\lambda \sqrt{-\mu }\right),\\ & v_{3}(\xi )=a_{0}+2\beta \sqrt{-\mu }\left(\coth\left(\xi_{0}+\sqrt{-\mu }\xi \right)+\lambda \sqrt{-\mu }\right)+R,\\ & v_{4}(\xi )=a_{0}+2\beta \sqrt{-\mu }\left(\tanh\left(\xi_{0}+\sqrt{-\mu }\xi \right)+\lambda \sqrt{-\mu }\right)+R, \end{array}$$

where *ξ*=*x*+*y*−(2*βμ*λ+2*βαμ*λ−*a*_0_−*αR*−*αa*_0_)*t*.

$$\begin{array}{rl} \mathrm{Family}\;3:\; & u_{5}(\xi )=a_{0}+4\beta \sqrt{-\mu }\coth\left(2\left(\xi_{0}+\sqrt{-\mu }\xi \right)\right),\\ & v_{5}(\xi )=a_{0}+4\beta \sqrt{-\mu }\coth\left(2\left(\xi_{0}+\sqrt{-\mu }\xi \right)\right)+R, \end{array}$$

where *ξ*=*x*+*y*+(*a*_0_+*αR*+*αa*_0_)*t*.

$$\begin{array}{rl} \mathrm{Family}\;4:\; & u_{6}(\xi )=a_{0}+\beta \sqrt{-\mu }\left(\coth\left(\xi_{0}+\sqrt{-\mu }\xi \right)+\lambda \sqrt{-\mu }\mp \mathrm{csch}\left(\xi_{0}+\sqrt{-\mu }\xi \right)\right),\\ & u_{7}(\xi )=a_{0}+\beta \sqrt{-\mu }\left(\tanh\left(\xi_{0}+\sqrt{-\mu }\xi \right)+\lambda \sqrt{-\mu }\mp \mathrm{Isech}\left(\xi_{0}+\sqrt{-\mu }\xi \right)\right),\\ & v_{6}(\xi )=a_{0}+\beta \sqrt{-\mu }\left(\coth\left(\xi_{0}+\sqrt{-\mu }\xi \right)+\lambda \sqrt{-\mu }\mp \mathrm{csch}\left(\xi_{0}+\sqrt{-\mu }\xi \right)\right)+R,\\ & v_{7}(\xi )=a_{0}+\beta \sqrt{-\mu }\left(\tanh\left(\xi_{0}+\sqrt{-\mu }\xi \right)+\lambda \sqrt{-\mu }\mp \mathrm{Isech}\left(\xi_{0}+\sqrt{-\mu }\xi \right)\right)+R, \end{array}$$

where *ξ*=*x*+*y*−(*βμ*λ+*βαμ*λ−*a*_0_−*αR*−*αa*_0_)*t*.

$$\begin{array}{rl} \mathrm{Family}\;5:\; & u_{8}(\xi )=a_{0}+\beta \sqrt{-\mu }\left\{\tanh\left(\xi_{0}+\sqrt{-\mu }\xi \right)\mp \mathrm{sech}\left(\xi_{0}+\sqrt{-\mu }\xi \right)\right\},\\ & u_{9}(\xi )=a_{0}+\beta \sqrt{-\mu }\left\{\coth\left(\xi_{0}+\sqrt{-\mu }\xi \right)\mp \mathrm{Icsch}\left(\xi_{0}+\sqrt{-\mu }\xi \right)\right\},\\ & v_{8}(\xi )=a_{0}+\beta \sqrt{-\mu }\left\{\tanh\left(\xi_{0}+\sqrt{-\mu }\xi \right)\mp \mathrm{sech}\left(\xi_{0}+\sqrt{-\mu }\xi \right)\right\}+R,\\ & v_{9}(\xi )=a_{0}+\beta \sqrt{-\mu }\left\{\coth\left(\xi_{0}+\sqrt{-\mu }\xi \right)\mp \mathrm{Icsch}\left(\xi_{0}+\sqrt{-\mu }\xi \right)\right\}+R, \end{array}$$

where *ξ*=*x*+*y*+(*a*_0_+*αR*+*αa*_0_)*t*.

Consequently, for *μ*>0, we can obtain corresponding five families of plane periodic solutions (which are omitted for convenience).

**Note**

From the obtained solutions for PIB equations we observe that in family 1 and family 6, *μ*≠−1/λ^2^.

**Remark 3.2**

All the obtained results have been checked with Maple 13 by putting them back into the original equation. All results are correct.

## 4. Graphical illustration of some obtained solutions

Graphical illustrations of some obtained solutions are shown in figures 1–8.

**Figure 1.**
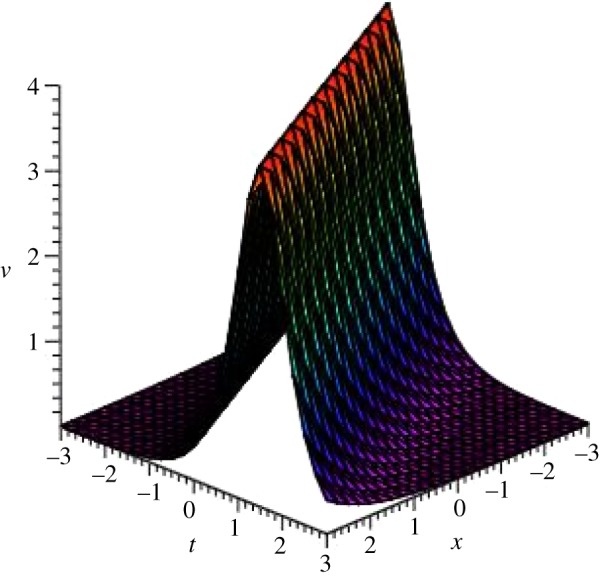
Bell-shaped soliton of *v*_3_(*ξ*) of DSW equation for particular values of *p*=1, *q*=2, *r*=*s*=1, *μ*=−0.25 and *ξ*_0_=0 within the interval −3≤*x*, *t*≤3.

**Figure 2.**
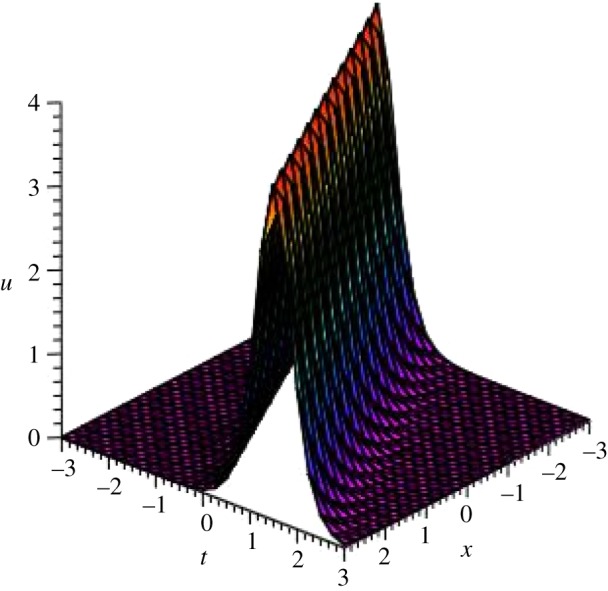
Bell-shaped soliton of *u*_3_(*ξ*) of DSW equation for particular values of *p*=1, *q*=2, *r*=*s*=1, *μ*=−0.25 and *ξ*_0_=0 within the interval −3≤*x*, *t*≤3.

**Figure 3.**
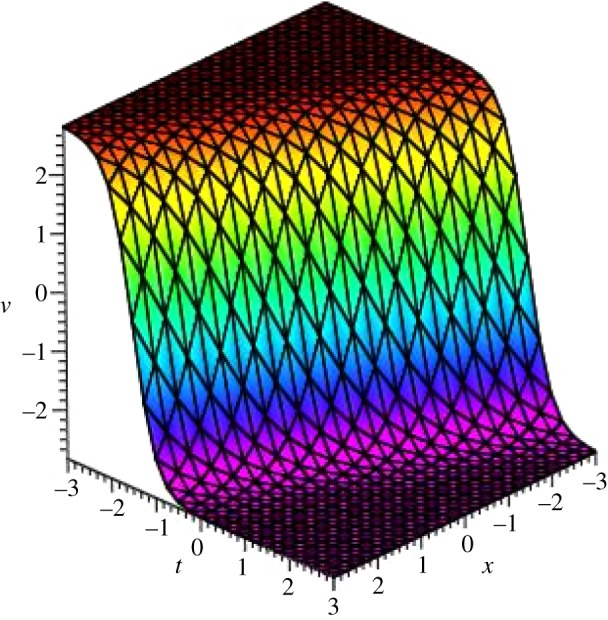
Kink profile of *v*_4_(*ξ*) of DSW equation for *p*=*q*=*r*=*s*=1, *μ*=−1 and *ξ*_0_=0 within the interval −3≤*x*, *t*≤3.

**Figure 4.**
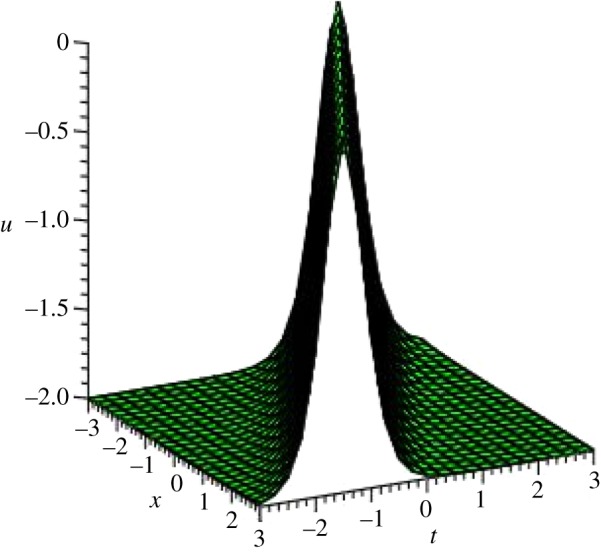
Bell-shaped soliton of *u*_4_(*ξ*) of DSW equation for *p*=*q*=*r*=*s*=1, *μ*=−1 and *ξ*_0_=0 within the interval −3≤*x*, *t*≤3.

**Figure 5.**
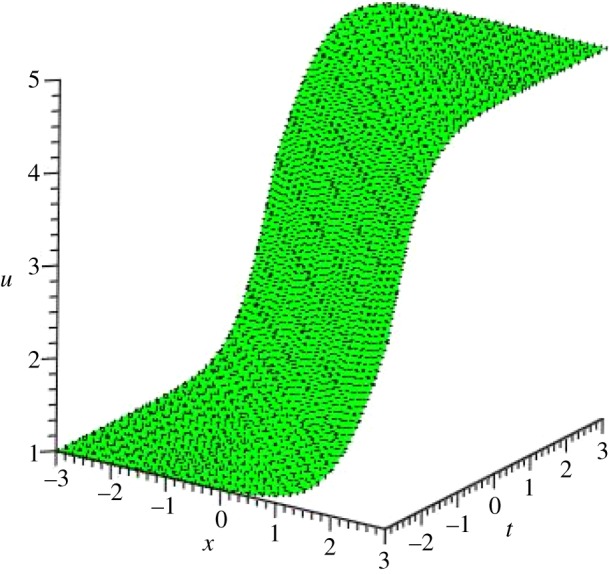
Kink profile of *u*_4_(*ξ*) of PIB equation for the values of *a*_0_=*α*=*β*=*γ*=1, *μ*=−1 and *A*=*y*=0 within the interval −3≤*x*, *t*≤3.

**Figure 6.**
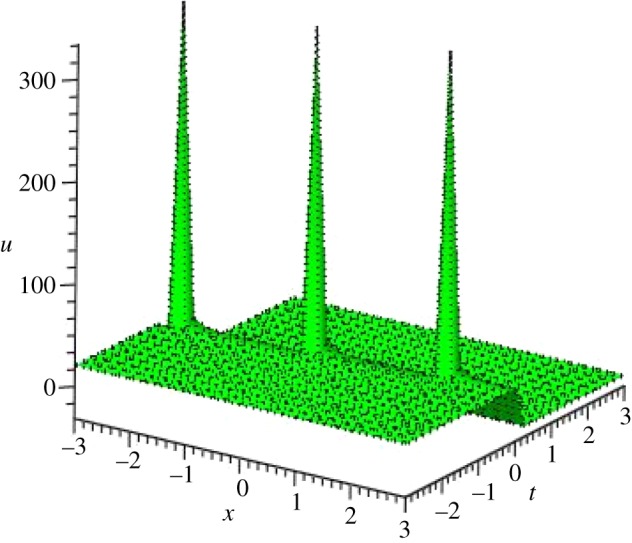
Kink profile of *v*_4_(*ξ*) of PIB equation for the values of *a*_0_=*α*=*β*=*γ*=1, *μ*=−1, *A*=*y*=0 and *R*=−5 within the interval −3≤*x*, *t*≤3.

**Figure 7.**
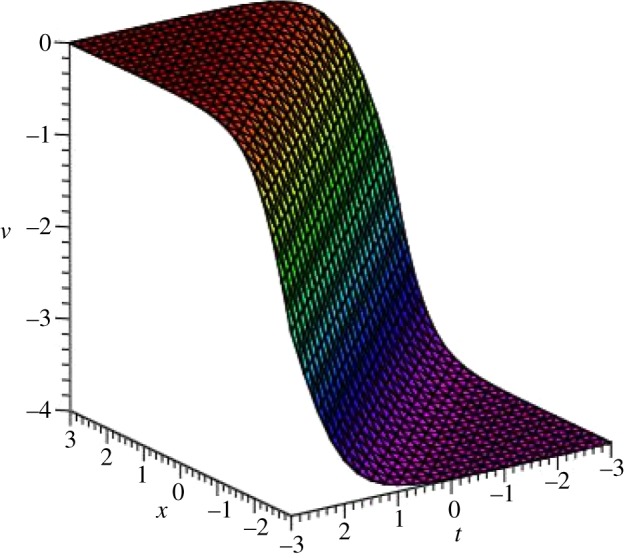
Singular soliton shape of *u*_5_(*ξ*) of PIB equation for the values of *a*_1_=0, *α*=*β*=λ=3, *μ*=−3, *y*=0 and *A*=−3 within the interval −3≤*x*, *t*≤3.

**Figure 8.**
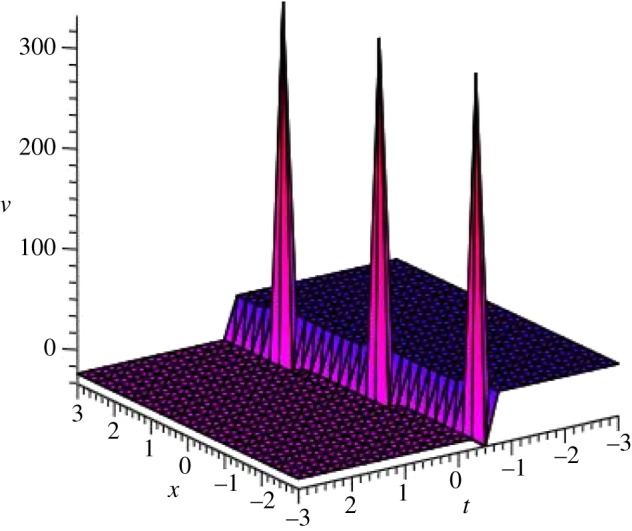
Singular soliton profile of *v*_5_(*ξ*) of PIB equation for the values of *a*_1_=0, *α*=*β*=λ=3, *μ*=−3, *y*=0 and *R*=*A*=−3, within the interval −3≤*x*, *t*≤3.

## 5. Conclusion

In this paper, we used the enhanced (*G*′/*G*)-expansion method to derive exact solutions with distinct physical structures, characterized by a novel solution, i.e. equation (2.4). This method with the help of symbolic computation software enabled us to construct broad new classes, *families*, of periodic and soliton solutions of the DSW equation and PIB equation. We have obtained some exact solutions of the DSW equation and PIB equation (involving parameters). When the parameters are taken as special values, the solitary wave solutions and the periodic solutions emerge from the exact solutions. It should be noted that the method used here can generate not only regular solutions but also singular ones involving csch and coth functions. The originality of the enhanced (*G*′/*G*)-expansion method is that equation (2.4) is not of the kind of series used usually in the applications of the modified method of simplest equation. Hence, the present approach extends the methodology of the modified method of simplest equation. This method including the structural characteristic equation (2.4) also applies to other types of nonlinear evolution equations, holding a promise to us for many more novel solutions. This advantage of the enhanced expansion method (EEM) stems from the best of two worlds: applying Kudryashov's simplest equation (2.5) under the assumption of rational solutions (*G*′/*G*).
